# Over-expression of an S-domain receptor-like kinase extracellular domain improves panicle architecture and grain yield in rice

**DOI:** 10.1093/jxb/erv417

**Published:** 2015-10-01

**Authors:** Xiaohua Zou, Zhengrui Qin, Chunyu Zhang, Bin Liu, Jun Liu, Chengsheng Zhang, Chentao Lin, Hongyu Li, Tao Zhao

**Affiliations:** ^1^Institute of Crop Science, Chinese Academy of Agricultural Sciences, Beijing 100081, China; ^2^Tobacco Research Institute, Chinese Academy of Agricultural Sciences, Qingdao 266101, China; ^3^Department of Molecular, Cell & Developmental Biology, University of California, Los Angeles, CA 90095, USA

**Keywords:** Panicle architecture, rice, S-domain receptor-like kinase.

## Abstract

The S-domain receptor kinase (SRK) comprises a highly polymorphic subfamily of receptor-like kinases (RLKs) originally found to be involved in the self-incompatibility response in *Brassica*. Although several members have been identified to play roles in developmental control and disease responses, the correlation between SRKs and yield components in rice is still unclear. The utility of transgenic expression of a dominant negative form of *SRK*, *OsLSK1* (*Large spike S-domain receptor like Kinase 1*), is reported here for the improvement of grain yield components in rice. *OsLSK1* was highly expressed in nodes of rice and is a plasma membrane protein. The expression of *OsLSK1* responded to the exogenous application of growth hormones, to abiotic stresses, and its extracellular domain could form homodimers or heterodimers with other related SRKs. Over-expression of a truncated version of *OsLSK1* (including the extracellular and transmembrane domain of OsLSK1 without the intracellular kinase domain) increased plant height and improve yield components, including primary branches per panicle and grains per primary branch, resulting in about a 55.8% increase of the total grain yield per plot (10 plants). Transcriptional analysis indicated that several key genes involved in the GA biosynthetic and signalling pathway were up-regulated in transgenic plants. However, full-length cDNA over-expression and RNAi of *OsLSK1* transgenic plants did not exhibit a detectable visual phenotype and possible reasons for this were discussed. These results indicate that *OsLSK1* may act redundantly with its homologues to affect yield traits in rice and manipulation of *OsLSK1* by the dominant negative method is a practicable strategy to improve grain yield in rice and other crops.

## Highlight

Dominant negative form of *OsLSK1* (*Large spike S-domain receptor like Kinase 1*) leads to the improvement of yield components, resulting in significantly increased grain yield in rice.

## Introduction

A rapid increase in world human population and a reduction in agricultural resources has promoted many researchers to carry out more biotechnology research to increase grain yield in cereal crops. Rice (*Oryza sativa* L.) is one of the most important staple cereal crops as it feeds more than half of the world’s population. Grain yield is an essential agronomic trait of rice which consists of many components including panicles per plant, grains per panicle, and grain weight (Xing and Zhang, 2010). To date, a number of genes have been reported to regulate this complex trait.

Plant receptor-like protein kinases (RLKs) comprise one of the largest and most diverse superfamily of plant proteins with 610 and 1 131 members in the *Arabidopsis* and rice genomes, respectively (Shiu *et al.*, 2004). The *RLK* gene superfamily has been implicated in the prevention of self-pollination, the pathogen defence response, hormone perception, developmental regulation, adaptation to abiotic stresses, and quantitative yield components (Haderlein *et al.*, 2001; Bai *et al.*, 2009; Lehti-Shiu *et al.*, 2009; Den Herder *et al.*, 2012). Plant RLKs are composed of an extracellular N-terminal domain which acts as a ligand-binding site, a single membrane-spanning region, and a conserved C-terminal cytoplasmic protein kinase domain (Castells and Casacuberta, 2007). According to the structural feature of the extracellular domain, RLKs are classified into 44 subfamilies, such as leucine rich repeat (LRR), self-compatibility domain (S-domain), wall-associated kinase (WAK), lectin (Shiu and Bleecker, 2001; Tor *et al.*, 2009), and many more. Among these subclasses one or only a small number of proteins have been characterized in detail.

The first plant RLK gene (*ZmPK1*) was isolated in maize (Walker and Zhang, 1990), and thereafter many RLK genes with diverse functions have been identified in many plant species (Becraft, 2002). For example, In the WAK RLK subfamily, WAK is required for cell expansion throughout plant development (Wagner and Kohorn, 2001). The function of the Lectin RLK protein, Pi-d2, is to enhance resistance to the rice blast fungus (Chen *et al.*, 2006). The calmodulin-binding receptor-like protein kinase (CBRLK1) acts as a negative regulator of pathogen resistance in *Arabidopsis* (Kim *et al.*, 2009). In the largest LRR RLK subfamily, CLAVATA1 (CLV1) controls meristem development (Clark *et al.*, 1993). Rice *Xanthomonas resistance 21* (*Xa21*) and *Xa26* are associated with enhanced tolerance to *Xanthomonas oryzae* pv*.oryzae* (Lee *et al.*, 2003; Wang *et al.*, 2006). BR insensitive 1 (BRI1) and BRI1 associated kinase 1 (BAK1) work together to perceive and modulate BR signaing (Li and Chory, 1997). HAESA is related to floral organ abscission (Jinn *et al.*, 2000); flagellin sensitive 2 (FLS2) can recognize the bacterial elicitor flagellin (Danna *et al.*, 2011; Gómez-Gómez and Boller, 2000); AvrPphB susceptible 1 (PBS1) confers resistance to *Pseudomonas syringae* strains (Swiderski and Innes, 2001); ERECTA1 regulates organ shape (Shpak *et al.*, 2003; van Zanten *et al.*, 2009). In addition, LRR-RLK subfamily can affect the yield components. Over-expression the LRR-RLK gene LRK1 improves yield components of rice, including the panicles per plant, spikelets per panicle, grains per plant, 1000-grain weight and plant height (Zha *et al.*, 2009).

S-domain RLKs (SRKs) represent the second largest subfamily of RLKs, with 147 members in rice. The well-characterized *Brasssica* S-domain RLK (SRK) is a self-incompatibility receptor which auto-phosphorylated when pollinated with incompatible pollen (Goring and Rothstein, 1992; Takasaki *et al.*, 2000; Cabrillac *et al.*, 2001). Expression pattern analysis showed that many genes encoding S-domain RLKs were broadly expressed in many tissues and rapidly accumulated in response to wounding and pathogen invasion, suggesting possible roles in both developmental control and disease responses (Dwyer *et al.*, 1994; Pastuglia *et al.*, 1997, 2002). The newly identified member *OsSIK2* increases stress tolerance and delays dark-induced leaf senescence in transgenic rice plants (Chen *et al.*, 2013).

In this study, an S-domain RLK *OsLSK1* associated with abiotic stress sensitivity and increased grain yield was characterized. Over-expression of the extracellular domain of OsLSK1, rather than the full length, improved the yield components in rice. The OsLSK1 extracellular domain can form dimers with itself or with five of the most homologous SRKs, suggesting ectopic expression of the extracellular domain of *OsLSK1* may cause a dominant negative effect to alter the yield components in rice. Further investigation showed that the GA biosynthetic and signalling pathway genes may be involved in the improvement of the yielding traits. Our study provides a new approach for yield improvement in cereal crops.

## Materials and methods

### Plant materials and growth conditions

Rice plants were cultivated at the Experimental Station of the Chinese Academy of Agricultural Sciences in Beijing (39°54′ N, 116°23’ E) during the summer. The field test experiments were performed at two locations with different soil fertility levels. Each location consisted of three replicates and each replicate included 10 individuals for each material. The relevant agronomical traits were analysed at the heading and mature stages. Statistical analysis was performed with independent samples using the least significance difference (LSD) software.

### Generation of transgenic rice plants

To generate the *OXOsLSK1-t* and *OXOsLSK1*-*f* vectors, the extracellular domain (1–1 590bp) and the full length of *OsLSK1* were cloned into the gateway entry vector pDONR 201 and then recombined into the pCAMBIA1301-Bar-FLAG vector, driven by the ubiquitin promoter. To construct the RNAi vector, a 309bp fragment (from 380–689bp) of *OsLSK1* was ampliﬁed and cloned into the gateway entry vector pDONR 201, and then recombined into pANDA vector. The primer sequences are listed in Supplementary Table S1 at *JXB* online. All constructs were introduced into *Agrobacterium tumefaciens* strain *EHA105* and then transformed into Kitaake wild-type plants (Gao *et al.*, 2013).

### GUS histochemical staining

To obtain *pOsLSK1::GUS* transgenic plants, a 2 249bp promoter region of *OsLSK1* was ampliﬁed using the forward primer 5′-TATTTTCGGTACAATGGAGGTCG-3′ and hte reverse primer 5′-CGTTTCAACTATAGCAGTTTGGC-3′ from the genome of Nipponbare, and inserted into the *Hin*dIII and *Bam*HI sites of the pCAMBIA3301-GUS vector. GUS histochemical staining assays were performed according to the method of Jefferson *et al.* (1987) and Laubinger *et al.* (2006).

### RNA isolation and qRT-PCR analysis

RNA was isolated using TRIZOL (Invitrogen) and treated with DNase I (Invitrogen). The cDNA was synthesized from 3.0 μg total RNA using *TransScript* ^®^ II One-Step gDNA Removal and cDNA Synthesis SuperMix (TransGen Biotech). LightCycler 480 SYBR Green I Master (Roche) was used for the quantitative PCR reaction. The mRNA level of *Actin* was used as the internal control. All the primers used are listed in Supplementary Table S1 at *JX*B online.

### Hormone treatments and abiotic stresses

To investigate the response of *OsLSK1* mRNA to hormone and abiotic stresses, the 3-week-old rice seedlings (Kitaake) were treated with 20 μM GR-24, 20% PEG, 1% H_2_O_2_, 200mM NaCl, and 0.1mM ABA, GA, BR, ABA, and JA as described in previously (Tang *et al.*, 2012; Chu *et al.*, 2013). Leaves samples were collected at 0, 2, 4, 8, 12, 24, 36, and 48h after the treatment. The *OsLSK1* expression was monitored by qRT-PCR. *Actin* was used as the internal control.

### Immunoblots

One-week-old seedlings of the over-expression lines and the wild-type controls were ground to extract protein. Immunoblots analysis were performed essentially as described by Meng *et al.* (2013).

### Subcellular localization of *OsLSK1*

Full-length *OsLSK1* was amplified and cloned into the gateway entry vector pDONR 201 and then fused in-frame at both the N- and C-terminus of YFP in the Gateway system (Invitrogen) vector pENSG-YFP and pEXSG-YFP under the control of the 35S CaMV promoter (Laubinger *et al.*, 2006). The *YFP*-*OsLSK1* and the *OsLSK1*-*YFP* constructs were transiently expressed in *Arabidopsis* leaf protoplasts (Abel and Theologis, 1994). The protoplasts were examined under a Leica TCS-SP4 confocal microscope after 12h incubation at 30 °C in the dark. The fluorescent lipophilic styryl dye FM4-64 was used to label the plasma membrane (Fischer-Parton *et al.*, 2000). Empty vectors were used as a control.

### Yeast two-hybrid assay

The yeast two-hybrid assay was performed according to the manufacturer’s instructions (ProQues two-hybrid system with Gateway technology, Invitrogen). The *OsLSK1* extracellular domain was fused in-frame to the GAL4 DNA binding domain in the bait vector pDEST32 (Invitrogen). The extracellular domain of *OsLSK1*, *LOC_Os01g47810*, *LOC_Os01g47820*, *LOC*_*Os01g48000*, *LOC*_*Os01g48020*, and *LOC_Os01g48040* were fused in-frame to the GAL4 DNA transcription activation domain in the prey vector pDEST22 (Invitrogen). The bait and prey plasmids were co-transformed into the yeast strain AH109 and grown on Trp-/Leu- plates at 28 °C for 2–3 d before being transferred to SD/Trp-/Leu-/His-/Ade plates at 28 °C for 2–3 d. The β-Gal activity was analysed by a colony-lift filter assay, using X-gal (5-bromo-4-chloro-3-indolyl-β-d-galacto-pyranoside) for blue colour development, according to the Yeast Protocols Handbook (Clontech). The yeast two-hybrid assay primers are listed in Supplementary Table S1 at *JXB* online.

### BiFC assays

The N-terminal (including the extracellular domain and the transmembrane domain) of *OsLSK1*, *LOC_Os01g47810*, *LOC_Os01g47820*, *LOC*_*Os01g48000*, *LOC*_*Os01g48020*, and *LOC_Os01g48040* were cloned into the pSPYNE (R) 173 or the SPYCE(MR) vector (Waadt and Kudla, 2008) and then transferred into *Agrobacterium tumefaciens.* The BiFC experiments were performed using *Nicotiana benthamiana* leaves through *Agrobacterium tumefaciens* infiltration as described by Meng *et al.* (2013). The YFP fluorescence was excited by a 514-nm laser and captured at 523–600nm. The construct primers are listed in Supplementary Table S1 at *JXB* online.

## Results

### Rice *OsLSK1* encodes a typical S-domain receptor-like protein

**Fig. 1.**
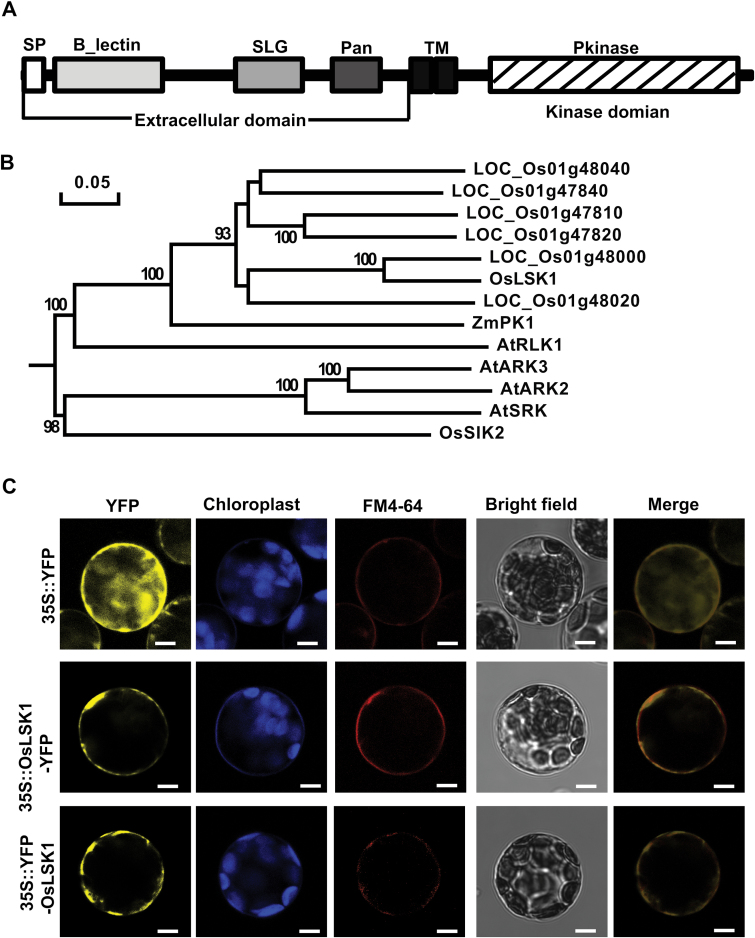
OsLSK1 encodes an S-domain RLK. (A) Schematic representation of the OsLSK1 protein. SP, signal peptide; SLG, S-locus glycoprotein; TM, transmembrane domain. (B) Rooted phylogenetic tree. The S-domain RLK sequences selected contain the conserve domain including B_Lectin, SLG(S locus glycoprotein), and Pan in the extracellular region. The amino acid sequences were aligned using Clustal X, and the phylogenetic tree was constructed by MEGA5.1 using the Neighbor–Joining method and bootstrap support was based on 1 000 replicates. (C) Subcellular localization of OsLSK1. The OsLSK1-YFP and YFP-OsLSK1 fusion proteins were mainly located in the plasma membrane. YFP was used as a control. Bar=5 μm.

The complete sequence of *OsLSK1* (2 460bp, GENBANK accession no: NM_001050355 GI: 115439080) was isolated and cloned by a reverse transcriptase-polymerase chain reaction (RT-PCR) from *Oryza sativa* ssp. *japonica* cv*. Nipponbare* seedlings. *OsLSK1* encodes a protein of 819 amino acid residues with a predicted molecular mass of 89.9kDa. Protein analysis of the deduced amino acid sequence revealed that OsLSK1 contains a typical Type III S-receptor kinase structure (Zhang *et al.*, 2011), including a B-Lectin domain (amino acids 76–188), an SLG domain (S_locus_glycop domain) (amino acids 238–313), a PAN like domain (amino acids 347–404), a transmembrane domain (amino acids 435–485), and a kinase domain at the C-terminal cytoplasmic region (amino acids 524–805) (Fig. 1A). Further analysis showed that the extracellular region had a membrane localization signal peptide composed of 27 amino acids at the N terminal; the intracellular domain contained the typical ATP-binding motif and a kinase active site (see Supplementary Fig. S1 at *JXB* online).

Amino acid alignment analysis showed that *OsLSK1* shares high similarity at both the intercellular and the extracellular domains with the homologues in rice (*OsSIK2*, *LOC_Os01g47810*, *LOC_Os01g47820*, *LOC*_*Os01g48000*, *LOC*_*Os01g48020*, and *LOC_Os01g48040*), maize (*ZmPK1*), and *Arabidopsis* (*AtRLK1*, *AtSRK*, *AtARK2*, and *AtARK3*) (see Supplementary Fig. S1 at *JXB* online). Phylogenetic analysis showed that *OsLSK1* shares 55–81% high identity with rice homologues, 73% identity with the maize homologue (*ZmPK1*), and only about 33% identity with the *Arabidopsis* genes (Fig. 1B).

The membrane localizing signal peptide and transmembrane domain of the OsLSK1 protein suggest that it may be a membrane protein. To test this, two constructs harbouring the fusion proteins YFP-OsLSK1 and OsLSK1-YFP were generated, in which YFP was fused to the 5′ and 3′ ends of the OsLSK1 protein, respectively. Transient expression in the *Arabidopsis* mesophyll protoplasts showed that both YFP-OsLSK1 and OsLSK1-YFP were exclusively located on the plasma membrane, co-localizing especially with the plasma membrane fluorescent lipophilic styryl dye FM4-64. By contrast, the control protein YFP was only detectable in the intracellular region (Fig. 1C). Together, these data suggest that OsLSK1 is a plasma membrane-localized protein.

### OsLSK1 forms dimers at the extracellular domain

**Fig. 2.**
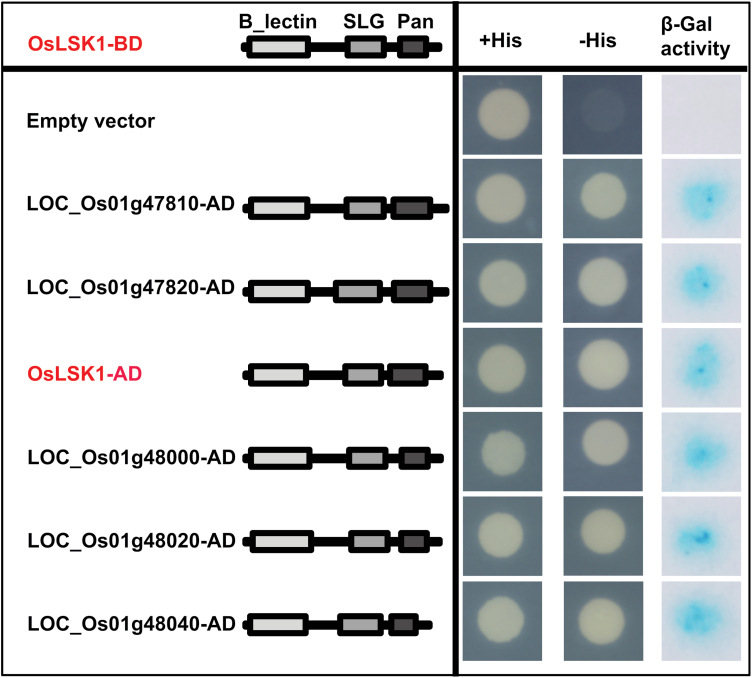
OsLSK1 extracellular domain interacts with itself and its homologues in yeast. A histidine autotrophy assay and a colony-lift filter assay showed that the constructs (left panel) were also able to transactivate the expression of the lacZ reporter genes (right panel). The homologues include *LOC_Os01g47810*, *LOC_Os01g47820, LOC_Os01g48000*, *LOC_Os01g48020*, and *LOC_Os01g48040*.

Rice genome annotation analysis (http://rice.plantbiology.msu.edu/index.shtml) showed that *OsLSK1* is located on chromosome 1 and clustered with other six homologous genes within a 130kb region, which was separated by ten retrotransposons (see Supplementary Fig. S2B at *JXB* online). A previous report indicated that the PAN-like domain in the SRK extracellular region can serve as the interaction part of the molecule (Naithani *et al.*, 2007). As shown in Fig. 1A, and in Supplementary Fig. S2A at *JXB* online, both *OsLSK1* and its six homologues in rice contained the PAN-like domain. This prompted us to investigate whether OsLSK1 interacts with itself or with other homologues. cDNAs of the extracellular region of *OsLSK1* and five homologues in rice (*LOC_Os01g47840* could not be cloned) were cloned into two yeast expression vectors (pDEST22 and pDEST32), respectively, and co-transformed into a yeast strain for the yeast two-hybrid experiment. Results showed that the extracellular domain of OsLSK1 could interact with itself and also with all five homologues (Fig. 2).

**Fig. 3.**
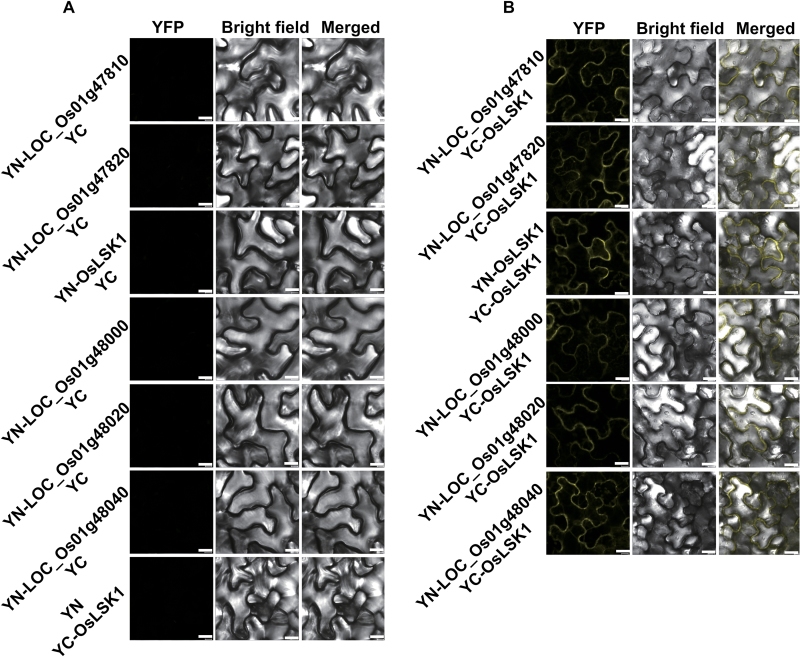
OsLSK1 extracellular domain interacts with itself or its homologues *in planta*. Epiﬂuorescence and bright-ﬁeld images of *N. benthamiana* epidermal leaf cells (scale bars=20 μm).

This result was then confirmed *in planta* using a bimolecular ﬂuorescence complementation (BiFC) assay. The extracellular domain (including the transmembrane domain) of OsLSK1 and its five homologues were fused with the N-terminal of YFP (YN) or the C-terminal of YFP (YC) and co-expressed in *N. benthamiana* leaves using the *Agrobacterium*-mediated infiltration method. Strong fluorescence signals were detected in the *N. benthamiana* epidermal cell plasma membrane which was co-transfected with *Agrobacterium tumefaciens* strains harbouring *YC-OsLSK1/YN-OsLSK1, YC-OsLSK1/YN-LOC_Os01g47810, YC-OsLSK1/YN-LOC_Os01g47820, YC-OsLSK1/YN-LOC_Os01g48000, YC-OsLSK1/YN-LOC_Os01g48020* or *YC-OsLSK1/YN-LOC_Os01g48040*. By contrast, the control which was co-transfected with *YC/YN-OsLSK1, YC/YN-LOC_Os01g47810, YC/YN-LOC_Os01g47820, YC/YN-LOC_Os01g48000, YC/YN-LOC_Os01g48020* or *YC/YN-LOC_Os01g48040* exhibited no signal (Fig. 3). Both the yeast two-hybrid and BiFC analysis indicated that OsLSK1 could interact with itself and also with five homologues, suggesting that OsLSK1 can form homodimers (maybe polymers) with itself and heterodimers with five homologues, respectively.

### Expression pattern analysis of *OsLSK1*

**Fig. 4.**
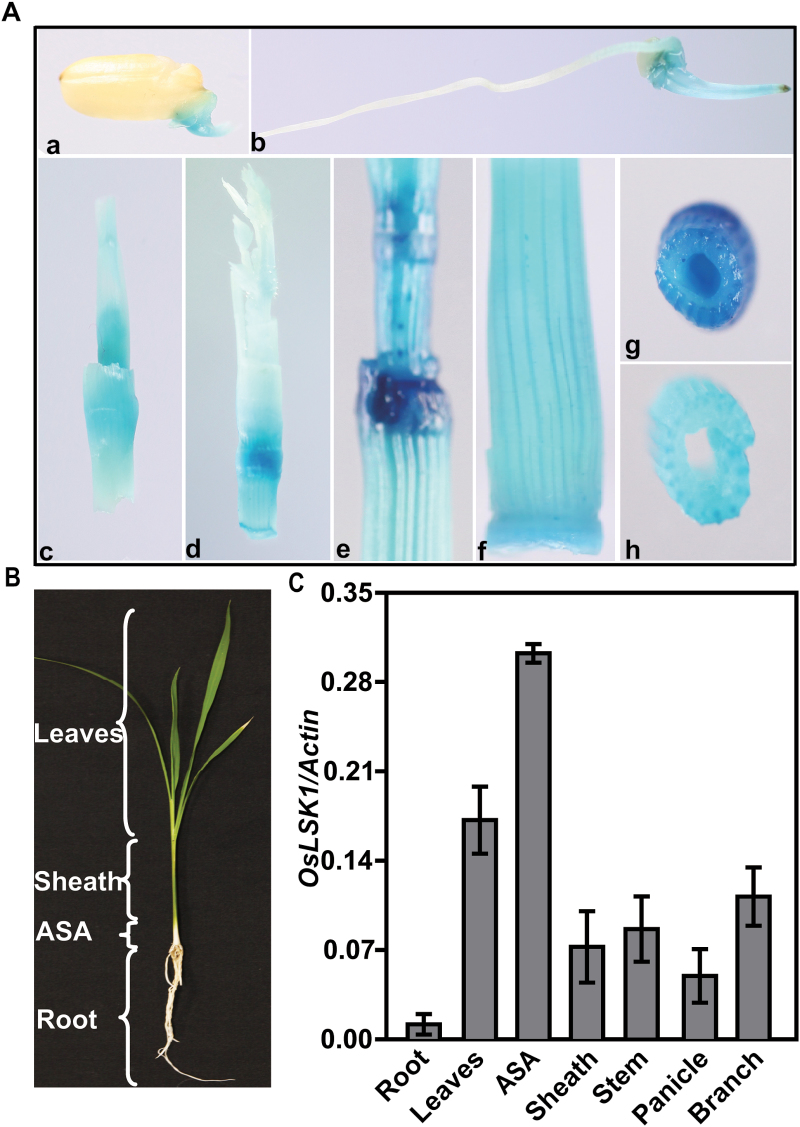
*OsLSK1* expression pattern in rice. (A) *OsLSK1* expression revealed by GUS staining in *OsLSK1* promoter–GUS transgenic plants.(a) Germinated seed (2 d), (b) a small seedling(5 d), (c) a shoot tip, (d) a young panicle (1.5cm long), (e) a stem node, (f) a sheath, (g) a cross-section of the stem node, and (h) a cross-section of the sheath. (B) Schematic diagram showing the root, leaves, and ASA (around the shoot apex) of a 30-d-old seeding. (C) The transcript level of *OsLSK1* in various tissues and organs by quantitative RT-PCR. Data are shown as means ±SD (*n*=3). Root, leaves, and ASA (around the shoot apex) of a 30-d-old seeding and the stem (without the stem node), panicle (2cm length), branch (4cm length panicle without grains) of heading stage wild-type plants (Kitaake) were used.

To investigate the tissue-specific expression pattern of *OsLSK1*, rice transgenic *pOsLSK1::GUS* reporter lines were used, in which the 2 249bp *OsLSK1* promoter region is fused to the *GUS* gene (Shapiro and Zhang, 2001). As shown in Fig. 4A, histochemical staining revealed that *OsLSK1* is expressed in the plumule of the germinating seed, the coleoptile, leaf, node, and sheath, but not in the young spike or root. *OsLSK1* expression was also monitored from seeding to mature caryopses and our results showed an extremely high expression level of *OsLSK1* in the stem, especially in the nodes, suggesting *OsLSK1* may play important roles in node development. The *OsLSK1* mRNA expression pattern was also analysed using qRT-PCR. Similar to the GUS staining results, *OsLSK1* was expressed in nearly all the tissues but only weakly in roots and panicles. By contrast with the GUS staining results, *OsLSK1* was highly expressed in young leaves and ‘Around the Shoot Apex’ region (ASA) (Fig. 4B, C), rather than in the stem. The difference between qRT-PCR and GUS staining may be due to posttranscriptional regulation.

**Fig. 5.**
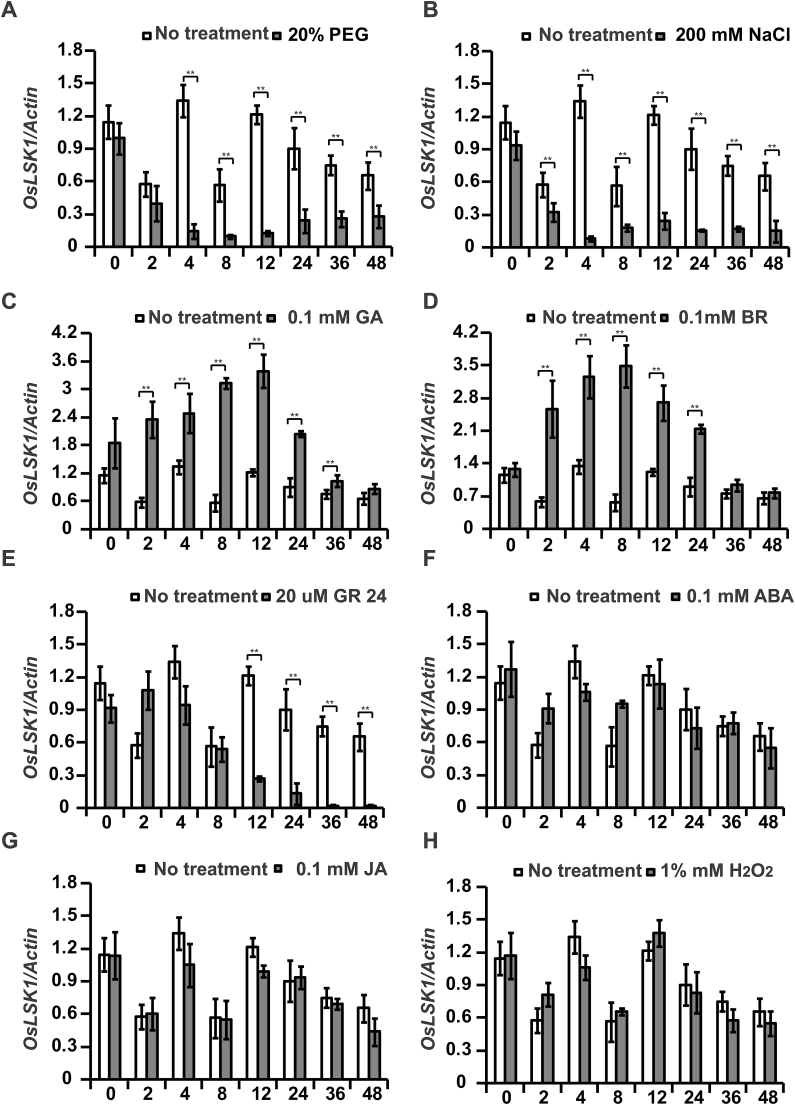
*OsLSK1* responds to hormones and abiotic stresses. Expression of *OsLSK1* in wild type (Kitaake) seedlings in response to (A) PEG, (B) NaCl, (C) gibberellin (GA), (D) brassinosteroid (BR), (E) strigolactone anologue GR 24, (F) ABA, (G) jasmonic acid (JA), and (H) H_2_O_2_ treatment by quantitative RT-PCR. Data are shown as means ±SD. (Student’s *t* tests, **P <0.01, *n*=3).

It has been reported that RLKs are transcriptionally regulated in response to hormone and abiotic stresses (Gish and Clark, 2011; Marshall *et al.*, 2012; Lin *et al.*, 2013; Wu and Zhou, 2013; Xu *et al.*, 2013). Therefore, expression of *OsLSK1* was also monitored under the phytohormone and abiotic stress treatments and the results indicated that *OsLSK1* responded differently to the various phytohormone treatments. *OsLSK1* was apparently induced by treatments with GA (0.1mM) and BR (0.1mM), with expression peaks at 4–8h after treatment. Thereafter, expression was gradually reduced (Fig. 5C, D). However, *OsLSK1* expression was inhibited by GR24 (20 μM) 8h after the treatment and reached the lowest level 48h later (Fig. 5E). In addition, no significant differences in *OsLSK1* expression were detected after the ABA (0.1mM) and JA (0.1mM) (Fig. 5F, G) treatments. The responses of *OsLSK1* to abiotic stresses such as polyethylene glycol (PEG) and salinity (NaCl) and H_2_O_2_ were also analysed. *OsLSK1* expression rapidly declined when treated with polyethylene glycol (PEG 20%) and NaCl (200mM) stress, but showed no obvious change when treated with H_2_O_2_ (1%) treatments (Fig. 5A, B, H).

### Over-expressing the extracellular domain of *OsLSK1* improves the quantitative yield components in rice

**Fig. 6.**
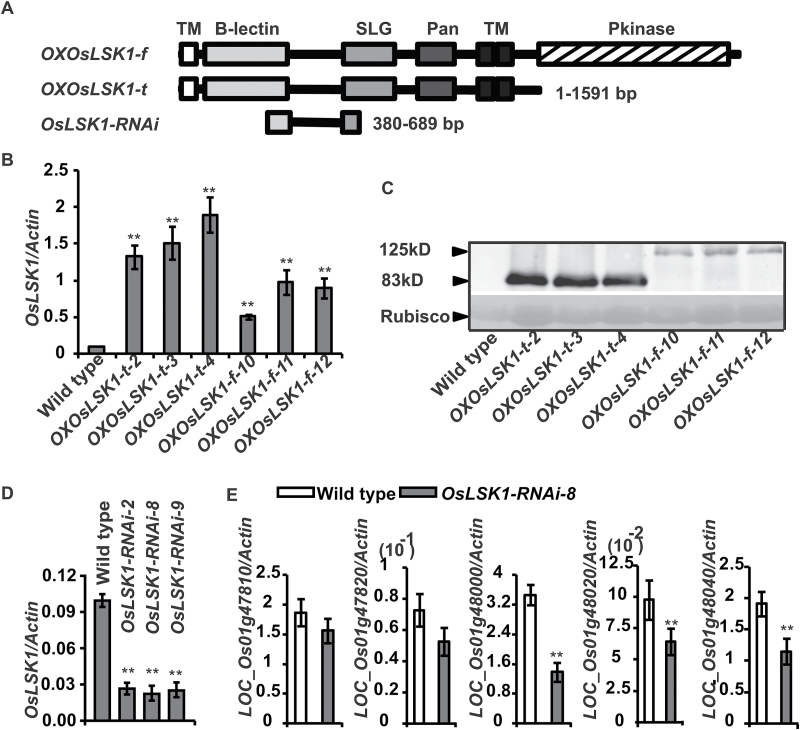
Identify of *OsLSK1* transgenic plants. (A) Schematic diagram showing the position of the *OsLSK1* transgenic construct including ‘*OXOsLSK1-f*’, over-expressing full-length *OsLSK1*; ‘*OXOsLSK1-t*’, over-expressing the extracellular domain of *OsLSK1* and *OsLSK1-RNAi*. (B) The expression of *OsLSK1* determined by quantitative RT-PCR. (C) Immunoblots showing the level of OsLSK1-t-Flag fusion protein in *OXOsLSK1-t* plants and OsLSK1-f-Flag fusion protein in *OsLSK1-f* plants. (D) Transcriptional abundance of *OsLSK1* homologous genes in wild type and *OsLSK1-RNAi* transgenic lines. Data are shown as means ±SD. (Student’s *t* tests, **P <0.01, *n*=3).

**Fig. 7.**
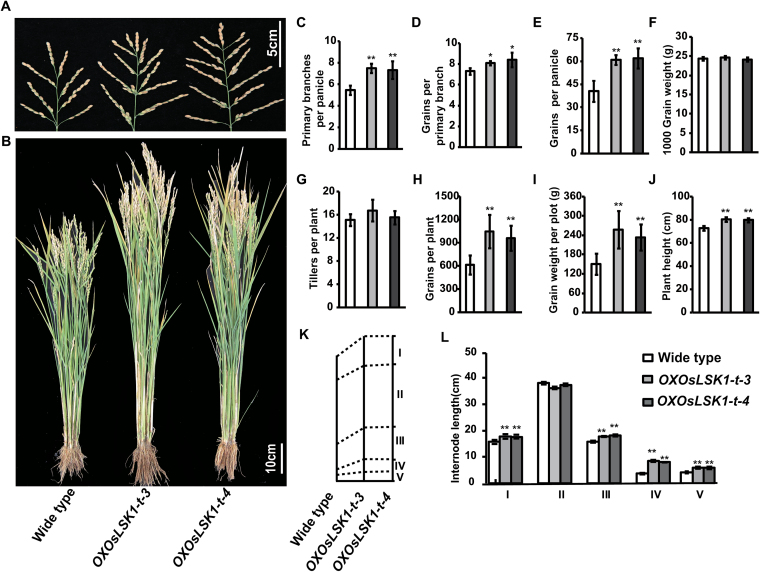
Phenotype analysis of OsLSK1-t over-expressing plants. (A) Panicle morphology of wild type and *OXOsLSK1-t*. (B) Gross morphology of wild-type and *OXOsLSK1-t* transgenic rice. (C–I) Comparison of (C) primary branches per panicle, (D) grains per primary branch, (E) grains per panicle, (F) 1 000 grain weight, (G) tillers per plant, (H) grains per plant, (I) grain weight per plot, and (J) plant height between the wild type and *OXOsLSK1-t* transgenic rice. Data are shown as means ±SD. (Student’s *t* tests, *P <0.05, **P <0.01, *n*=60). (K, L) Schematic representation (K) and comparison of the various elongation patterns of internodes (L) in the wild type and *OXOsLSK1* transgenic rice. The first to fifth internodes are indicated as I–V from top to bottom. Data are shown as means ±SD. (Student’s *t* tests, **P <0.01, *n*=10).

To examine the biological function of *OsLSK1*, multiple independent lines of T_0_ transgenic rice were obtained by *Agrobacterium*-mediated transformation, in which the full-length gene (*OsLSK1-f*) and the truncated gene (*OsLSK1-t*) (Fig. 6A) were over-expressed, respectively. Molecular analysis results showed that both *OsLSK1*-*f* and *OsLSK1-t* were highly expressed at both the RNA and protein levels in most of the transgenic lines tested (Fig. 6B, C). In addition, *OsLSK1-RNAi* T_0_ transgenic rice was also obtained. qRT-PCR results showed that *OsLSK1* was dramatically reduced in most of the T_0_ *OsLSK1-RNAi* lines (Fig. 6D). The expression of the *OsLSK1* homologues was also examined. Among them, *LOC_Os01g48000* mRNA was reduced to a significantly low level, while others were only slightly down-regulated (Fig. 6E). Phenotype analysis indicated that *OXOsLSK1-t*, but not the *OXOsLSK1-f* and *OsLSK1-RNAi* transgenic plants, showed obvious phenotypes during the entire rice growth period compared with wild-type rice (see Supplementary Fig. S3 at *JXB* online). Thereafter, two lines (*OXOsLSK1-t-3* and *OXOsLSK1-t-4*) which accumulated the highest expression levels of RNA and protein in all of the transgenic lines tested were selected for further study (Fig. 7).

Compared with the wild-type plant, *OXOsLSK1-t-3* and *OXOsLSK1-t-4* showed larger panicles, higher grain yields, and taller culm phenotypes (Fig. 7). To analyse the larger panicle phenotype, the branching pattern of *OXOsLSK1-t-3* and *OXOsLSK1-t-4* was examined in more detail. On average, *OXOsLSK1-t-3* and *OXOsLSK1-t-4* panicles possessed 7.50 and 7.33 primary branches, respectively, while the wild-type panicle only possessed 5.48 primary branches. This indicates that the primary branches per panicle were significantly more abundant in *OXOsLSK1-t-3* and *OXOsLSK1-t-4* than in the wild type (Fig. 7C). The number of grains produced on the primary branches (Fig. 7D) was also determined. The results showed that the number of grains per primary branch of the *OXOsLSK1-t-3* and *OXOsLSK1-t-4* transgenic lines were 8.11 and 8.42, respectively, significantly higher than that of the wild type (7.33) (Fig. 7D). As a result, the increase in primary branches and grains produced on the primary branches led to the dramatic increase in grain number per panicle in *OXOsLSK1-t-3* and *OXOsLSK1-t-4* which were 60.84 and 61.80, respectively, whereas it was only 40.46 in wild-type plants (Fig. 7E).

Other components of the rice yield trait were also examined such as tiller number, seed setting rate (SSR), and 1000-grain weight. Statistical analysis revealed that there was no difference between *OXOsLSK1-t-3*, *OXOsLSK1-t-4*, and wild-type plants (see Supplementary Table S2 at *JXB* online; Fig. 7F, G) for seed setting rate (SSR), 1000-grain weight, and tiller number. The actual grain yield at plot level (10 plants per plot) was also measured; the results showed that the grain yield of *OXOsLSK1-t-3* and *OXOsLSK1-t-4* increased more than 55.8% compared with wide-type plants (Fig. 7G). The increased grain number per panicle appears to explain why the *OXOsLSK1-t-3* and *OXOsLSK1-t-4* plants have a higher grain yield.

To investigate culm phenotype, internode length at a late stage of rice growth was measured. It was found that all the internodes of *OXOsLSK1-t-3* and *OXOsLSK1-t-4*, except for the second (II) internode, were significantly increased compared with that of the wild type. This increase in internode length was particularly obvious in the fourth and fifth internodes (Fig. 7J, K, L).

### GA biosynthetic and signalling pathway genes were up-regulated in the *OXOsLSK1-t* plant

**Fig. 8.**
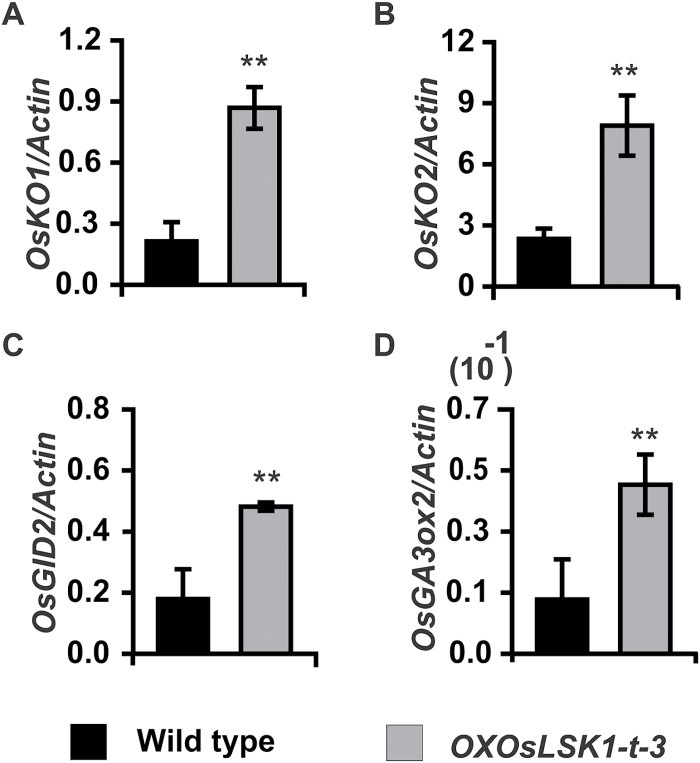
Quantitative RT-PCR analysis of plant morphologically related genes in *OsLSK1-t* over-expressing plants. (A): *OsKO1*, (B): *OsKO2*, (C): *OsGID2*, (D): *OsGA3ox2*. Data are shown as means ±SD. (Student’s *t* tests, **P <0.01, *n*=3).

Our data indicated that *OsLSK1* is positively responsive to the GA and BR treatments, which suggests that the GA and BR biosynthetic or signalling pathways may associate with the phenotype of the *OXOsLSK1-t* transgenic plants. To test this speculation, qRT-PCR was used to compare the mRNA expression of several key genes involved in the GA (*OsKO1*, *OsKO*, and *GA20ox2*) or BR (*OsBRI1* and *OsGSR1*) biosynthesis and GA signal transduction pathways (*OsGID2*) between *OXOsLSK1-t-3* and wild-type seedlings at the six-leaf stage. qRT-PCR results showed that the expression of genes (*OsKO1*, *OsKO2*, *GA20ox*, and *OsGID2*) involved in the GA biosynthesis and signalling transduction pathways were significantly up-regulated (2–4-fold) in *OXOsLSK1-t* transgenic seedlings compared with their respective expression in the wild type (Fig. 8). However, there were no significantly different expression changes observed for the BR biosynthesis genes between the wild-type and *OXOsLSK1-t* transgenic seedlings (see Supplementary Fig. S4 at *JXB* online). These data suggest that the phenotypes of *OXOsLSK1-t* transgenic plants might be related to the expression changes of genes involved in the GA biosynthetic and signalling pathways.

## Discussion

Rapid population growth has made food shortage a serious problem in the world, prompting more studies on biotechnology to improve grain yield in crops. Rice grain yield is mainly determined by three quantitative component traits, including panicle per plant, grains per panicle, and grain weight (Bai *et al.*, 2009; Xing and Zhang, 2010). Grains per panicle is a highly variable trait and depends on the structural features of the panicle including the number of primary and secondary branches, panicle length, and the percentage of filled grains. Although several genes have been reported to regulate these traits, the gene networks that control rice yield remained elusive. In this study, an S-domain receptor-like kinase gene, *OsLSK1*, is reported which localizes in the plasma membrane and is broadly expressed in various tissues and organs (Figs 1, 4). Over-expression of the *OsLSK1* extracellular domain affects various pleiotropic phenotypes such as plant height, primary branch number, grain number per primary branch, and grain number per panicle, resulting in a significant increase in the grain yield per plant (Fig. 7; see Supplementary Fig. S3 at *JXB* online).

The plant hormone GA includes a large group of tetracyclic diterpenoid compounds and regulates diverse biological processes such as seed germination, flowering, and plant height (Sun, 2008). Physiological studies and phenotypic characterization of dwarf mutants indicated that GA determines plant height primarily through increased internode elongation (Sun, 2008; Salas Fernandez *et al.*, 2009). A number of studies also show that the exogenous application of GA could lead to significantly increases in various growth characters, namely, plant height, number of tillers, grains per plant,and yield attributes in rice (Pan *et al.*, 2013). Our data proved that *OsLSK1* was up-regulated under high concentrations of GA. Moreover, over-expression of the extracellular domain of *OsLSK1* showed a similar phenotype to that of the exogenous application of GA. Further investigations showed that *OsKO1*, *OsKO2*, *GA20ox2*, and *OsGID2* were up-regulated in the *OXOsLSK1-t* plant. *OsKO1* and *OsKO2* are ent-kaurene oxidases and encode the key branch-point enzyme involved in the first step of GA biosynthesis. *GA20ox2* is a gibberellin 20-oxidase, a key oxidase enzyme in the biosynthesis of gibberellin that catalyses the conversion of GA12 and GA53 to GA9 and GA20, respectively. *OsGID2* (*GA-insensitive dwarf2*) is a component of the SCF complex which mediates GA-dependent DELLA protein degradation to positively regulate gibberellin (GA) signalling. These results suggested that the phenotype of *OXOsLSK1-t* transgenic plants might be caused by both increasing the endogenous GA concentration and by enhanced GA signal transduction.

**Fig. 9.**
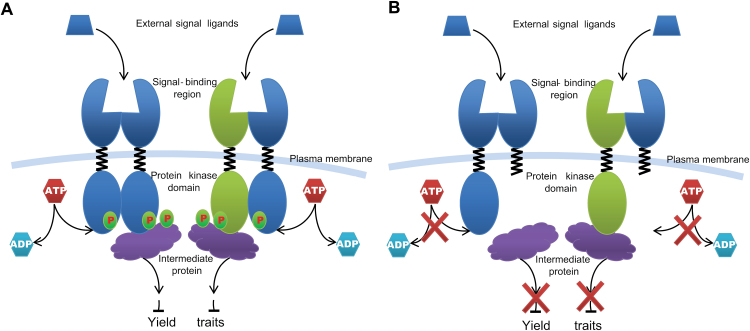
A working model of *OsLSK1.* (A) A working model of RLKs. (B) Dominant negative working model of RLKs.

Receptor-like kinase are one of the largest protein families in plants with over 1000 members in rice (Shiu *et al.*, 2004). In general, a RLK contains three functional domains: an extracellular domain, a transmembrane domain, and an intracellular serine/threonine kinase domain. The activation of RLKs is phosphorylation-dependent. External signal ligands are recognized by the extracellular domain which triggers phosphorylation activity of the intracellular cytoplasmic kinase domain. The intracellular cytoplasmic kinase domain then phosphorylates substrate proteins within the cell which activates the downstream signalling pathways. In this process, the dimerization of RLKs is required for its function (Stokes and Gururaj Rao, 2008) (Fig. 9A). For example: the *Arabidopsis* *CRINKLY4*, is functional as a homodimer to control development of the integument and seed coat (Stokes and Gururaj Rao, 2008); the formation of a heterodimer between BRI1 and BAK1 is critical for their function in BR signal transduction(Nam and Li, 2002). The extracellular domain of the S-domain receptor kinase possesses a PAN-like domain, which was reported to be a protein interaction domain (Naithani *et al.*, 2007). The S-receptor kinase *OsLSK1* has six homologous genes in the rice genome, sharing 55– 81% identity. These seven S-domain RLK genes clustered within a 130kb region and were separated by ten retrotransposons (see Supplementary Fig. S2 at *JXB* online). The yeast two-hybrid and BiFC data showed that *OsLSK1* could form homo-/heterodimers with itself or its homologous proteins at the extracellular domain (Figs 2, 3). In this study, full-length *OsLSK1* over-expressing plants and RNAi plants did not show any visible phenotype. However, over-expression of a truncated version of *OsLSK1*, in which the intracellular kinase domains were deleted, improved the plant height and yield components. It is proposed that dimerization of OsLSK1 and its homologous proteins could underlie these surprising observations (Fig. 9B). When the truncated versions of the OsLSK1 proteins were introduced into plants, they formed the functionless heterodimers with endogenous OsLSK1 and its homologues to block the phosphorylation activity of the intracellular cytoplasmic kinase domain, therefore disrupting external signal transduction. In the meantime, the non-functional *OsLSK1-t* homodimers and heterodimers compete with the internal functional OsLSK1 homodimers and heterodimers for the external signal ligands which are required for signal transduction. As a consequence, the truncated versions of *OsLSK1* exhibited dominant negative phenotypes that positively affect the yield components in transgenic rice.

The expression levels of *OsLSK1* and its homologues were examined in RNAi plants. Although the expression of *OsLSK1* was dramatically decreased in RNAi transgenic lines, the homologous genes were not severely affected. The functional redundancy might explain why no phenotype was observed in *OsLSK1-RNAi* transgenic lines (see Supplementary Fig. S3 at *JXB* online). For the full-length *OsLSK1* transgenic plants, there are two possibilities: one is that there were insufficient signal molecules for the excess exogenous and endogenous *OsLSK1* proteins to show the phenotype; the other is that the appropriate trigger of the phenotype was not found. Fortunately, a new approach has been found by using the over-expression of the extracellular domain of *OsLSK1* to improve the yield components in rice.

## Supplementary data

Supplementary data can be found at *JXB* online.

Supplementary Fig. S1. Amino acid sequence alignment of the OsLSK1 homologous proteins.

Supplementary Fig. S2. The protein structures and genome distribution of OsLSK1 homologous genes.

Supplementary Fig. S3. Phenotype analysis of OXOsLSK1-f and OsLSK1-RNAi plants.

Supplementary Fig. S4. Quantitative RT–PCR analysis of plant morphologically related genes in OXOsLSK1-t plants.

Supplementary Table S2. Primers.

Supplementary Table S2. Yield traits of transgenic plants.

Supplementary Data
