# Supplementary material for: Over-expression of an S-domain receptor-like kinase extracellular domain improves panicle architecture and grain yield in rice
Source: J Exp Bot. 2015 Oct 1;66(22):7197–209. doi: 10.1093/jxb/erv417 (PMC4765790; doi:10.1093/jxb/erv417)
Supplement: Supplementary Data [file supp_66_22_7197__index.html]

Over-expression of an S-domain receptor-like kinase extracellular domain improves panicle architecture and grain yield in rice — Over-expression of an S-domain receptor-like kinase extracellular domain improves panicle architecture and grain yield in rice — Supplementary Data 

# Over-expression of an S-domain receptor-like kinase extracellular domain improves panicle architecture and grain yield in rice

## Supplementary Data

Data files

- Supplementary Data - Supplementary Data
