# Supplementary material for: Over-expression of an S-domain receptor-like kinase extracellular domain improves panicle architecture and grain yield in rice
Source: J Exp Bot. 2015 Oct 1;66(22):7197–209. doi: 10.1093/jxb/erv417 (PMC4765790; doi:10.1093/jxb/erv417)
Supplement: Supplementary Data [file supp_erv417_Supplementary_data.pdf]

Figure legend

Supplementary Fig. S1. Amino acid sequence alignment of the OsLSK1 homologous proteins*.* *LOC_Os01g47810, LOC_Os01g47820, LOC_Os01g47840, OsLSK1 (LOC_Os01g47900), LOC_Os01g48000, LOC_Os01g48020, LOC_Os01g48040* and *OsSIK2* are from rice, *ZmPK1* is from maize, *AtRLK1* (*AT5G60900*), *AtARK1* (*AT1G65790*), *AtARK3* (*At4G21380*) and *AtSRK* are from *Arabidopsis*. The black region shows the some amino acids. The grey color indicates relative conservative region.

Supplementary Fig. S2. The protein structures and genome distribution of *OsLSK1* homologous genes. (A): The conserved domain structure diagram in OsLSK1 and six homologous. **(B):** *OsLSK1* cluster with six homologous genes in chromosome 1. The *OsLSK1* was labeled with red circle.

Supplementary Fig. S3. Phenotype analysis of *OXOsLSK1-f* and *OsLSK1-RNAi* plants. (A): The panicle morphologies of wild-type, *OXOsLSK1-f* and *OsLSK1-RNAi* transgenic rice. **(B):** The gross morphologies of wild-type, *OXOsLSK1-f* and *OsLSK1-RNAi* transgenic rice. **(C), (E):** Comparison of plants height, primary branch number between wild-type and *OXOsLSK1-f* transgenic rice. **(D), (F):** Comparison of plants height, primary branch number between wild-type and *OsLSK1-RNAi* transgenic rice**.** Data are shown as means ± s.d. (Student's *t* tests, $*P < 0.05$, $**P < 0.01$, n=60).

Supplementary Fig. S4. Quantitative RT–PCR analysis of plant morphologies related genes in *OXOsLSK1-t* plants. Data are shown as means ± s.d. (Student's *t* tests, $**P < 0.01$, n=3).

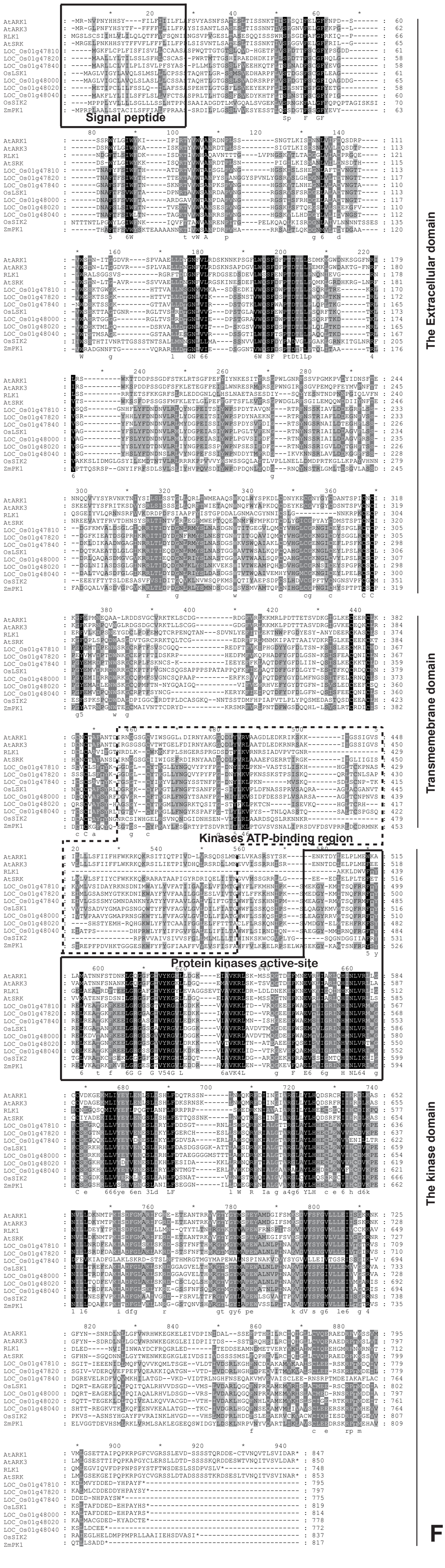

Signal peptide
Kinases ATP-binding region
Protein kinases active-site
The Extracellular domain
Transmembrane domain
The kinase domain
AtARK1
AtARK3
RLK1
AtSRK
LOC_Os01g47810
LOC_Os01g47820
LOC_Os01g47840
OsLSK1
LOC_Os01g48000
LOC_Os01g48020
LOC_Os01g48040
OsSIK2
ZmPK1

Figure S1

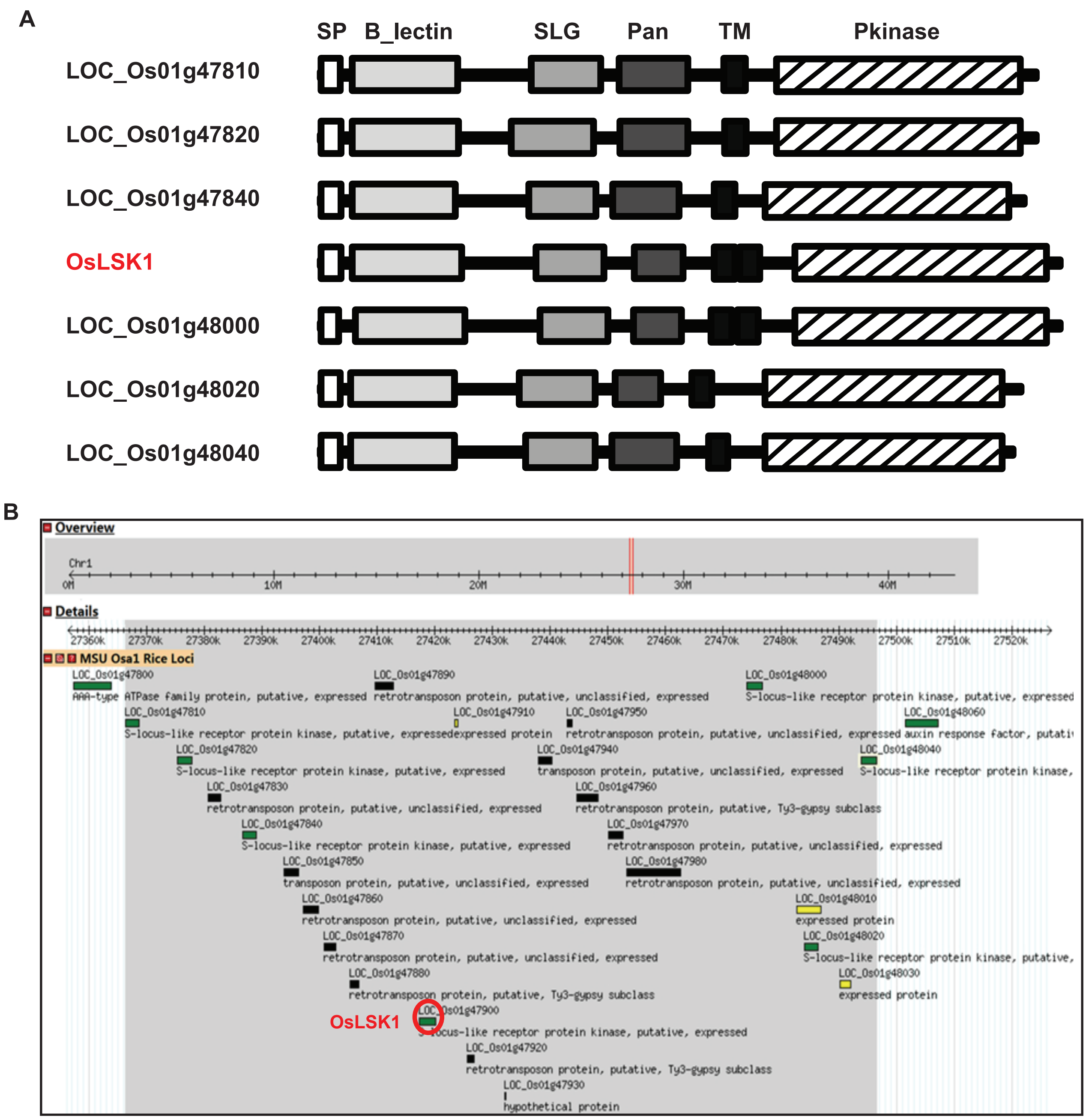
A
SP
B_lectin
SLG
Pan
TM
Pkinase
LOC_Os01g47810
LOC_Os01g47820
LOC_Os01g47840
OsLSK1
LOC_Os01g48000
LOC_Os01g48020
LOC_Os01g48040
B
Overview
Chr1
0M
10M
20M
30M
40M
Details
27360k
27370k
27380k
27390k
27400k
27410k
27420k
27430k
27440k
27450k
27460k
27470k
27480k
27490k
27500k
27510k
27520k
MSU Osa1 Rice Loci
LOC_Os01g47800
AAA-type ATPase family protein, putative, expressed
LOC_Os01g47890
retrotransposon protein, putative, unclassified, expressed
LOC_Os01g48000
S-locus-like receptor protein kinase, putative, expressed
LOC_Os01g47810
S-locus-like receptor protein kinase, putative, expressed
LOC_Os01g47910
expressed protein
LOC_Os01g47950
retrotransposon protein, putative, unclassified, expressed
LOC_Os01g48060
auxin response factor, putati
LOC_Os01g47820
S-locus-like receptor protein kinase, putative, expressed
LOC_Os01g47940
transposon protein, putative, unclassified, expressed
LOC_Os01g48040
S-locus-like receptor protein kinase,
LOC_Os01g47830
retrotransposon protein, putative, unclassified, expressed
LOC_Os01g47960
retrotransposon protein, putative, Ty3-gypsy subclass
LOC_Os01g47840
S-locus-like receptor protein kinase, putative, expressed
LOC_Os01g47970
retrotransposon protein, putative, unclassified, expressed
LOC_Os01g47850
transposon protein, putative, unclassified, expressed
LOC_Os01g47980
retrotransposon protein, putative, unclassified, expressed
LOC_Os01g47860
retrotransposon protein, putative, unclassified, expressed
LOC_Os01g48010
expressed protein
LOC_Os01g47870
retrotransposon protein, putative, unclassified, expressed
LOC_Os01g48020
S-locus-like receptor protein kinase, putative,
LOC_Os01g47880
retrotransposon protein, putative, Ty3-gypsy subclass
LOC_Os01g48030
expressed protein
OsLSK1
LOC_Os01g47900
S-locus-like receptor protein kinase, putative, expressed
LOC_Os01g47920
retrotransposon protein, putative, Ty3-gypsy subclass
LOC_Os01g47930
hypothetical protein

Figure S2

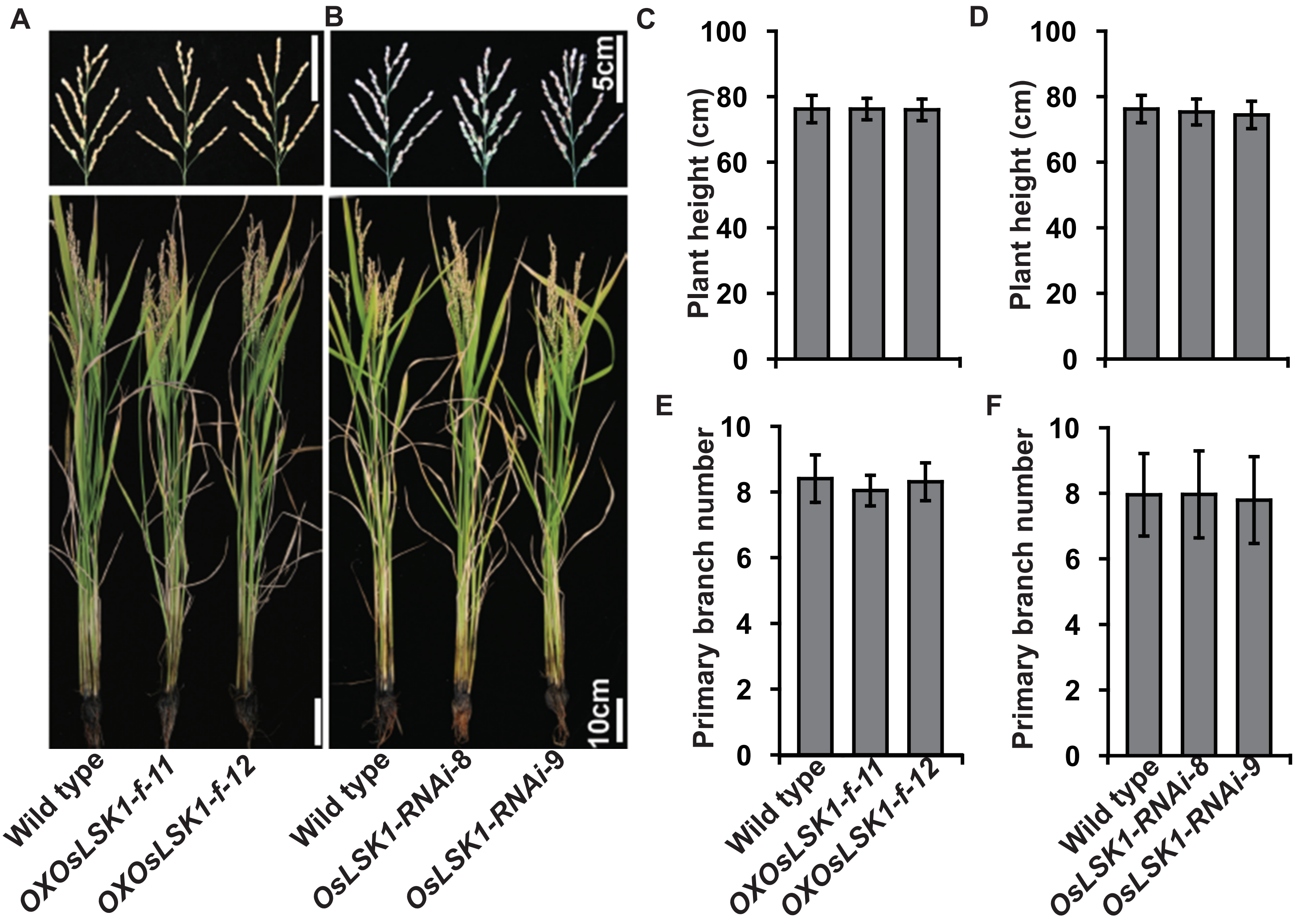
A
B
5cm
10cm
Wild type
OXOsLSK1-f-11
OXOsLSK1-f-12
Wild type
OsLSK1-RNAi-8
OsLSK1-RNAi-9
C
D
Plant height (cm)
0
20
40
60
80
100
E
F
Primary branch number
0
2
4
6
8
10

Figure S3

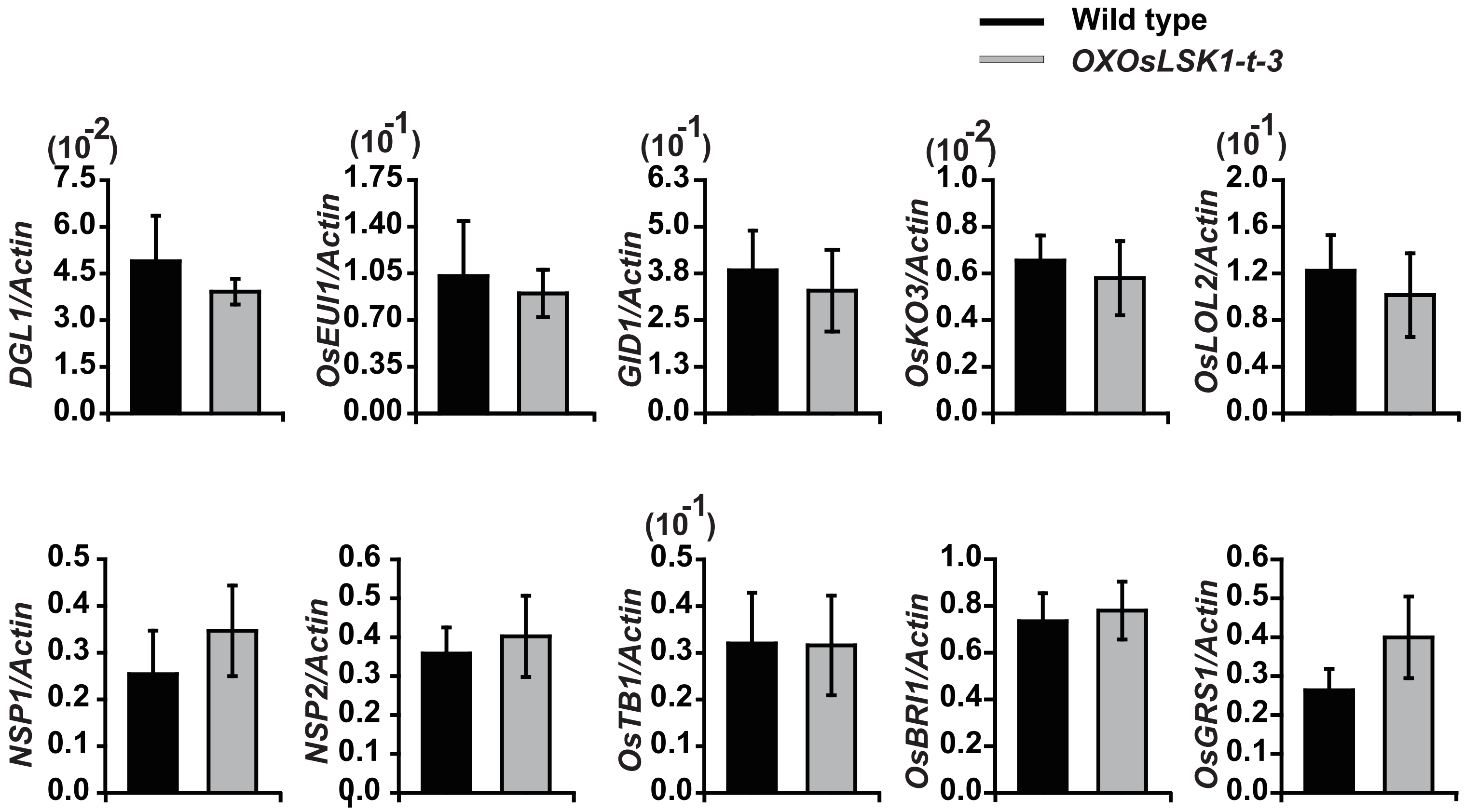

Wild type
OXOsLSK1-t-3
DGL1/Actin
OsEUI1/Actin
GID1/Actin
OsKO3/Actin
OsLOL2/Actin
NSP1/Actin
NSP2/Actin
OsTB1/Actin
OsBRI1/Actin
OsGRS1/Actin

Figure S4

Table S1 Primers

	Gene	Forward primer	Reverse primer
Vector construction for transgenic plant	OsLSK1-t	CAAAAAAGCAGGCTTCATGGCTGGATTGGTCATCG	CAAGAAAGCTGGGTCTCGATGAACTTGAACT
	OsLSK1-f	CAAAAAAGCAGGCTTCATGGCTGGATTGGTCATCG G	CAAGAAAGCTGGGTCAGAGTGGTAGGCAGGGTGTT
	OsLSK1-GUS	TATTTTCGGTACAATGGAGGTCG	CGTTTCAACTATAGCAGTTTGGC
	OsLSK1RNAi	CAAAAAAGCAGGCTTCGGGGCCTGACCGTCTCCCT	CAAGAAAGCTGGGTCACGCCGGCGTCGTCGAGGAT
Vector construction for Yeast two-hybrid	LOC_Os01g47810	CAAAAAAGCAGGCTTCACAGGTACATCTCTCCAGGT	CAAGAAAGCTGGGTCATAAACCTGGCCATTGTAG
	LOC_Os01g47820	CAAAAAAGCAGGCTTCTCACACATCAGAGCTGAGGA	CAAGAAAGCTGGGTCTAAGCCTTTAGTGTAAC
	OsLSK1	CAAAAAAGCAGGCTTCCTGGGCAGATCCTCCATGT	CAAGAAAGCTGGGTCGAAGCCGTTGAACAGCGT
	LOC_Os01g48000	CAAAAAAGCAGGCTTCCAGCACACGCTCGGCACCG	CAAGAAAGCTGGGTCGTAGCCGTTGAAAAGCCT
	LOC_Os01g48020	CAAAAAAGCAGGCTTCTCATTCTTATCGGTAGAAG	CAAGAAAGCTGGGTCCCGGCCGTTGTAGAGCATA
	LOC_Os01g48040	CAAAAAAGCAGGCTTCACCGGCTCATCTTTCTCGG	CAAGAAAGCTGGGTCACCATTGAACAGGAGGCCT
	attB adaptor	GTGGGGACAAGTTTGTACAAAAAAGCAGGCTTC	GTGGGGACCACTTTGTACAAGAAAGCTGGGTC
Positive transgenic lines test	OsLSK1-1(for *OsLSK1RNAi*)	GGTGCTTACTCCTAATCATCAT	GTCATGTGTTGAAAAGTCTA
	OsLSK1-2(for *OsLSK1OX*)	ATGGCTGGATTGGTCAT	ACCGACATGGAGGATCT
	Actin	ATCCTTGTATGCTAGCGGTCGA	ATCCAACCGGAGGATAGCATG

Q-PCR	LOC_Os01g47810	CATTGACATCGGTAAAAGC	CATGCCACCGACAGGACGGTG
	LOC_Os01g47820	CCTGCAGCTACTGCATAAAT	TGCAATATAATCATTCCAATG
	LOC_Os01g48040	GACACTGATATGTGTGCCTA	CATATCTATTTGGCGGTGGATG
	LOC_Os01g48000	ATCACCAGGCTAACCGTTAGCA	TGAAGTCTTAACTACAATTT
	LOC_Os01g48020	CACTCATCCAGTACCACCA	TGGTGGTACTGGATGAGTG
	OsKO1	AGATGACCCACATGCTC	CATCCACATAGACTGAA
	OsKO2	TCAGCTGGGTTCCAAAC	CCACGCACAATCCTTTC
	GID2	ATGTCGCAGCCTGCTG	GCAACCTTCTCCATG
	OsGA3ox2	TAGCCGACGAGTTGCTGA	TACCAGTTGAGGTGC
	OsKO3	ATAGAGGCTGCAGATAC	CAATGAGCTGGAACAGAG
	GID1	GTACCTGGACAGGAG	TAGATGCGCACCTCCA
	DGL1	ACCACTGGATGAGTATC	GTACTAGATTGACCTCG
	OsGSR1	AGGTGCTCCAACACGCAG	TCCTTGGTCTTCCAGTTG
	OsEUI1	CAAGCCCAAGTACCTCCAGA	TGACACGGGCCATGTAGAA
	OsLOL2	AGTGTATCTCCAGTACTGG	CTCCCTGTACGTTACCTCCA
	Dwarf10	ACTATGGAACCTCGGC	ACTCGATCTGCCGGTGC
	Dwarf14	TCGCCAAGCTTGTC	CATCGCGTCGAACAC
	Dwarf27	TGTTGCTCGACGGTGAG	GTCTCCGTCTGCACCG
	HTD2	ATGCTGCGATCGACGC	ACCATCGTCTTCTCCG
	NSP1	ACTCCCACTACAACAATAA	TTGCCGCATTCTTCTAGGT
	NSP2	ACACTTGGAGAGTACGAGG	ATCCAACGCTCCACCCTCAC
	OsTB1	ATGGACATACCGCTTT	GTGGTGGTAGTAGAA
	OsBRI1	ATGGTGGGTGGATCTGG	TCCTTGACCGGCTCGCCT
	Dwarf1	ATGGGCTCATCCTGTAGCA	TGCATGGATAACTGATGTGT
	Dwarf88	TGCTGCGATCGACGCAT	CACCATCGTCTTCTC
	OsSPY	ATGGGGCGACCGGGGA	TCCTTCCCGTCCAACT
	OsLRK1	ATCTCTTGGCAACCTG	GACGATGAGTTTGCTG
	Ghd7	ATGGGGATGGCCAATGAG	CTGCTGGTCCCATCGACA
	MOC1	ATGCATGAGAGTGATG	ACCACAGCATGCAGAG

Vector construction for BiFC assay	pSPYNE (R)-LOC_Os01g47810	GCCTACTAGTGGATCCATGGGTAAGTTCCTGTGCCCAC	TCCTACCCGGGAGCGGTACCAAGAAAGTACCACCCTGTCATG
	pSPYNE (R)-LOC_Os01g47820	GCCTACTAGTGGATCCATGGCTGCGCTGCTGTATCTAAC	TCCTACCCGGGAGCGGTACCGGACTTGGGTATGTTATGCTTC
	pSPYNE (R)-OsLSK1	GCCTACTAGTGGATCCATGGCTGGATTGGTCATCGGCTAC	TCCTACCCGGGAGCGGTACCCTTGCTGGAGAGGAACCACCAG
	pSPYNE (R)-LOC_Os01g48000	GCCTACTAGTGGATCCATGGCTGGATTGGTCATCGGCTG	TCCTACCCGGGAGCGGTACCGCTCTGCTTGCTGGACAGGAACCA
	pSPYNE (R)-LOC_Os01g48020	GCCTACTAGTGGATCCATGGAAACAATTCCCTGCCTC	TCCTACCCGGGAGCGGTACCTGTAGCCTCCTTCAATTCTCTG
	pSPYNE (R)-LOC_Os01g48040	GCCTACTAGTGGATCCATGGCAAAGTTTCTCTATCTGATC	TCCTACCCGGGAGCGGTACCCCTTATGGAAAGAAACCACCAAGC
	pSPYCE(M)-OsLSK1	GCCTACTAGTGGATCCATGGCTGGATTGGTCATCGGCTAC	GAGCGGTACCCTCGAGCTTGCTGGAGAGGAACCACCAG

Table S2 Yield traits of transgenic plants.

	Primary branchs per panicle	Grains per primary branch	Grains per panicle	1000 Grain weight (g)	Tillers per plant	Grains per plant	Grain weight per plot (g)	Plant height (cm)	Seed setting rate
**Wild type**	5.48±0.42	7.33±0.31	40.46±6.88	24.36±0.51	15.12±1.03	611.46±123.64	149.83±32.76	72.85±2.17	0.93±0.02
**OXOsLSK1-t-3**	7.50±0.44 **	8.11±0.21 *	60.84±3.20 **	24.63±0.47	16.77±1.86	1049.29±217.26	257.82±58.91 **	80.54±2.02 **	0.92±0.02
**OXOsLSK1-t-4**	7.33±0.82 **	8.42±0.71 *	61.80±6.47 **	24.11±0.52	15.55±1.17	961.96±163.69 [illegible]	233.44±40.93 **	79.77±1.76 **	0.92±0.04

Data are shown as means ± s.d.(Student's *t* tests, * P < 0.05, ** P < 0.01, n=60).
